# Medicine in Context: ten years’ experience in diversity education for medical students in Greater Western Sydney, Australia

**DOI:** 10.3205/zma001314

**Published:** 2020-03-16

**Authors:** Brahmaputra Marjadi, Virginia Mapedzahama, Gayle Rogers, Margaret Donnelly, Anne Harris, Dale Donadel, Emilie Jakstas, Tinashe Dune, Winston Lo, Sowbhagya Micheal, Trelawny McKnight, Annemarie Hennessy, Vaishnavi Anu Ganapathy, Fiona Pacey

**Affiliations:** 1 Western Sydney University, School of Medicine, Campbelltown, Australia; 2 Western Sydney University, Translational Health Research Institute, Campbelltown, Australia; 3 The University of Sydney, Susan Wakil School of Nursing and Midwifery, Faculty of Medicine and Health, Sydney, Australia; 4 Northcott Disability Service, North Parramatta, Australia; 5 Myrtle Cottage Group Inc., Ingleburn, Australia; 6 Western Sydney Local Health District, Integrated and Community Health, Sydney, Australia; 7 Chester Hill Neighbourhood Centre, Chester Hill, Australia; 8 Western Sydney University, School of Health Science, Campbelltown, Australia; 9 Royal North Shore Hospital, St Leonards, Australia

**Keywords:** diversity, medical education, community participation, social accountability

## Abstract

**Objective: **This paper describes the Western Sydney University School of Medicine (WSUSoM) diversity education program, Medicine in Context (MiC). MiC implements community-engaged learning and partnership pedagogy in teaching diverse social determinants of health to first clinical year medical students. Central to MiC content and delivery methods is the local region’s diversity which is also reflected in the student population and MiC staff.

**Methodology: **This is a descriptive report about how the WSUSoM staff with community and General Practice (GP) partners have co-designed, co-delivered, co-assessed and co-evaluated the MiC program in 2009-2018. In keeping with the community-engaged learning and partnership pedagogy, the report is co-authored by a cross section of MiC stakeholders: the WSUSoM staff members, community partners and an alumna.

**Results: **Ten weeks' immersion in community-based services, with debriefing and scaffolding in tutorials and workshops, exposes students to the complex interplay between social determinants of health and clinical practice. Sharing of experiences, insights and reflections in safe environments enables students to overcome the uneasiness of diversity education. Quality assurance reviews identified positive trends in students’ quality of learning and satisfaction in the program following evidence-based continuous improvements of the program design and delivery.

**Conclusion: **Implementation of community-engaged learning and partnership pedagogy in the MiC program, supported by ongoing commitment from the WSUSoM and its community and GP partners, has been successful in engaging students in diversity education. The synthesis of diversity education and clinical learning throughout the MiC program is an important step toward building competency in patient-centred care.

## Zusammenfassung

**Zielsetzung: **In diesem Beitrag wird das Diversity-Ausbildungsprogramm an der Universität West-Sydney, Fachbereich Medizin (WSUSoM, Western Sydney University School of Medicine), Medizin im Kontext (MiK), beschrieben. Das MiK-Programm implementiert gemeinschaftsorientiertes Lernen und partnerschaftliche Pädagogik, indem Medizinstudenten in ihrem ersten klinischen Jahr die vielfältigen sozialen Gesundheitsdeterminanten vermittelt werden. Entscheidend für die MiK-Inhalte und Schulungsmethoden ist die Diversität (engl. Diversity) der lokalen Region, die sich auch in der jeweiligen Studentenpopulation und den MiK-Mitarbeitern widerspiegelt.

**Methodik: **In diesem Bericht wird beschrieben, wie die Mitarbeiter der WSUSoM das MiK-Programm in der Zeit von 2009-2018 gemeinsam mit Partnern aus der Gemeinschaft und Ärzteschaft (niedergelassene Allgemeinmediziner, GPs) konzipiert, vermittelt, bewertet und evaluiert haben. In Einklang mit dem Konzept des gemeinschaftsorientierten Lernens und der partnerschaftlichen Pädagogik wurde dieser Beitrag in Zusammenarbeit mit einem Querschnitt unterschiedlicher MiK-Akteure erstellt: den WSUSoM-Mitarbeitern, Partnern aus der Gemeinschaft sowie ehemaligen Absolventen (den Alumni).

**Ergebnisse:** Im Rahmen einer zehnwöchigen Phase im Gesundheits- & Pflegedienst mit anschließender Nachbereitung in Tutorials und Workshops lernen die Studenten das komplexe Zusammenspiel zwischen den sozialen Gesundheitsdeterminanten und der klinischen Praxis kennen. Durch den Austausch von Erfahrungen, Erkenntnissen und Gedanken in sicherer Umgebung können die Studenten ihre Berührungsängste im Zusammenhang mit der Diversity-Ausbildung überwinden. Nach kontinuierlichen evidenzbasierten Verbesserungen am Konzept und der Umsetzung des Programms in die Praxis wurden bei Qualitätssicherungskontrollen positive Trends hinsichtlich der Lernqualität und der Zufriedenheit der Studenten mit dem Programm festgestellt.

**Fazit: **Durch die Implementierung des gemeinschaftsorientierten Lernens und der partnerschaftlichen Pädagogik im MiK-Programm konnten die Studenten durch fortlaufendes Engagement der WSUSoM und ihrer Partner aus der Gemeinschaft und Ärzteschaft erfolgreich in die Diversity-Ausbildung eingebunden werden. Die Synthese aus Diversity-Ausbildung und klinischem Lernen während des gesamten MiK-Programms ist ein wichtiger Schritt zur Entwicklung von Kompetenzen für eine patientenorientierte Versorgung.

## 1. Background

There is an increasing body of work on the value and significance of diversity education in medical programs [1], [2], [3]. This paper adds to this body of work by discussing the implementation of diversity education at the Western Sydney University School of Medicine (WSUSoM), Australia. The paper outlines how the Medicine in Context (MiC) program at the WSUSoM exposes students to diversity as part of the social determinants of health and an integral part of patient-centred care. Discussion of four aspects of the MiC program (philosophy, pedagogy, clinical integration and evaluation) will be followed by MiC evolution as the WSUSoM transitions from a Bachelor of Medicine and Bachelor of Surgery (MBBS) course to a Doctor of Medicine (MD). This transition from a Bachelor’s to a Professional Master’s degree includes programmatic improvements in research skills teaching and the creation of a professional portfolio and a scholarly project. 

The WSUSoM was established in Greater Western Sydney (GWS) in 2007 to help develop the medical workforce for under-served communities in Australia starting from the GWS region [4]. The GWS region is one of the fastest growing and most diverse in Australia, and home to the largest urban Aboriginal and Torres Strait Islander communities [5]. Over 50% of its two million inhabitants are migrants or their descendants from more than 170 countries. Over 100 languages are spoken in GWS; 38% of the population and up to 90% in some suburbs speak a non-English language at home [6], [7], [8]. 

Since its inception, the WSUSoM adopted social accountability principles [9] and embedded community-engaged learning components throughout its curriculum [5]. Therefore, GWS diversity is considered from student selection through to curriculum design and content. The School has a preferential system for applicants who have been GWS residents for five years or more resulting in a student diversity which reflects that of the GWS population [10]. Most of Western Sydney University students are ‘first-in-family’ to undertake a university degree [10] and the WSUSoM was designed to take high-school leavers. However, to allow those for whom access to medical training had previously been unattainable, mature-aged students are also able to apply. Particular attention is given to supporting Aboriginal and Torres Strait Islander students and the WSUSoM is proud that these endeavours are making a significant contribution to national efforts to increase the numbers of Aboriginal and Torres Strait Islander doctors in order to address gross discrepancies in life expectancy for our Indigenous peoples ([11], pp.36-37). 

The WSUSoM has a 5-year fully-integrated curriculum which is centred on problem-based learning in Years 1-2 and immersive clinical learning in hospital and community settings in Years 3-5. The curriculum started as a Bachelor of Medicine/Bachelor of Surgery (MBBS) degree in 2007 at a time when all primary medical qualifications in Australia were thus provided, and is transitioning to a Doctor of Medicine (MD) degree in 2019-2023 as part of a national trend. 

**Figure 1:**
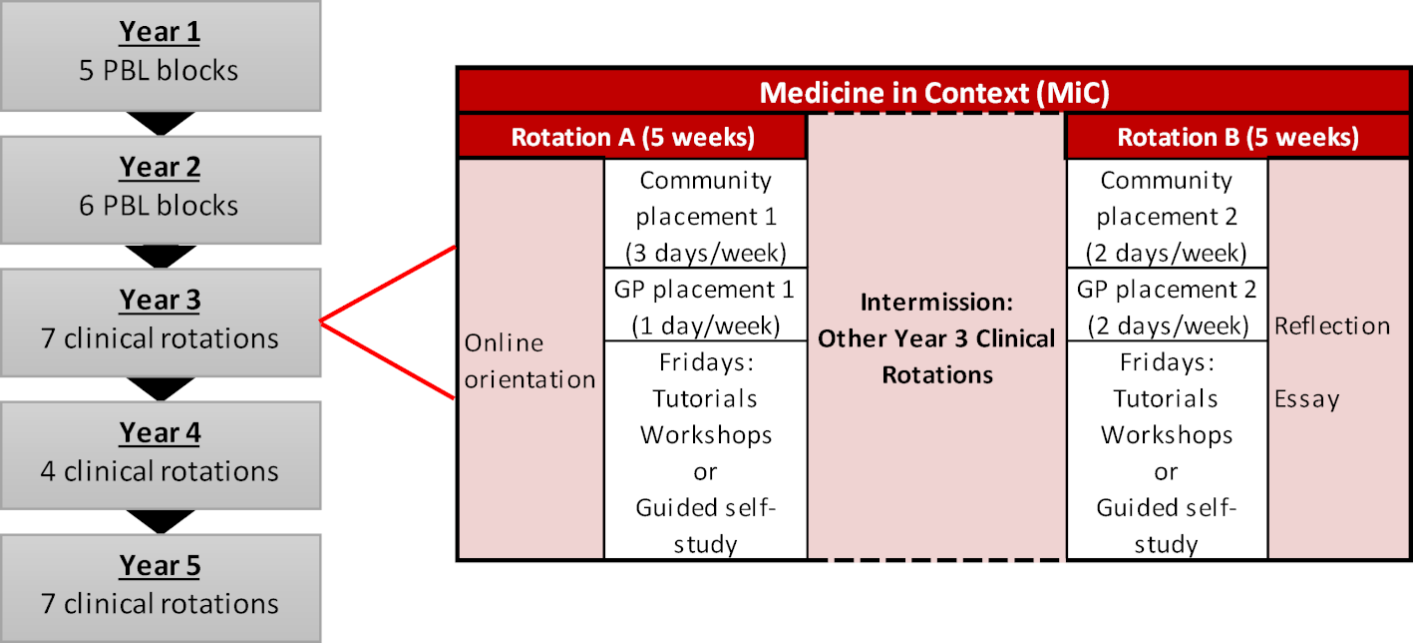
Medicine in Context program location and structure at the Western Sydney University School of Medicine MBBS curriculum.

The WSUSoM flagship diversity learning component is called Medicine in Context (MiC) and delivered in two 5-week rotations in the first clinical year (Year 3). Students attend a combination of community and General Practice (GP) placements for four days each week and return for campus-based tutorials and workshops every Friday to debrief and consolidate their placement learning experiences (see figure 1 (Fig. 1)). 

## 2. MiC program description

### 2.1. Philosophy

The MiC program was conceptualised after extensive community consultation [5] in line with the social accountability philosophy [9]. The program immerses first full clinical year students in community-based services including GP clinics to learn about the interplay between social determinants of health and clinical medicine. The MiC team implement the principles of community-engaged medical education [12] and partnership pedagogy [13] by involving community partners to contribute their wealth of experience in the community by co-designing, co-delivering, co-assessing and co-evaluating the program. 

#### 2.2. Pedagogy

The MiC program’s pedagogy has evolved through continuous evaluation and improvement. MiC design originally consisted of orientation, community and GP placements, tutorials, guest lectures and reflections [5]. These teaching methods have been continually improved using evidence-based approaches. Blended learning strategies including flipped classrooms and online evaluations are adopted to improve students’ engagement [14], [15] and scaffold increasing cognitive load [16]. Single-topic didactic lectures were replaced with interactive complex case discussions using games and role plays to introduce intersectionality between diversity aspects and facilitate a synthesis of community and clinical learning. MiC community and GP placements begin with a discussion between students and supervisors, where gaps in students’ life experience in regard to exposure to diversity are identified and inform the development of a placement learning plan that would facilitate their personal and professional growth.

##### 2.2.1. Community placements 

**Figure 2:**
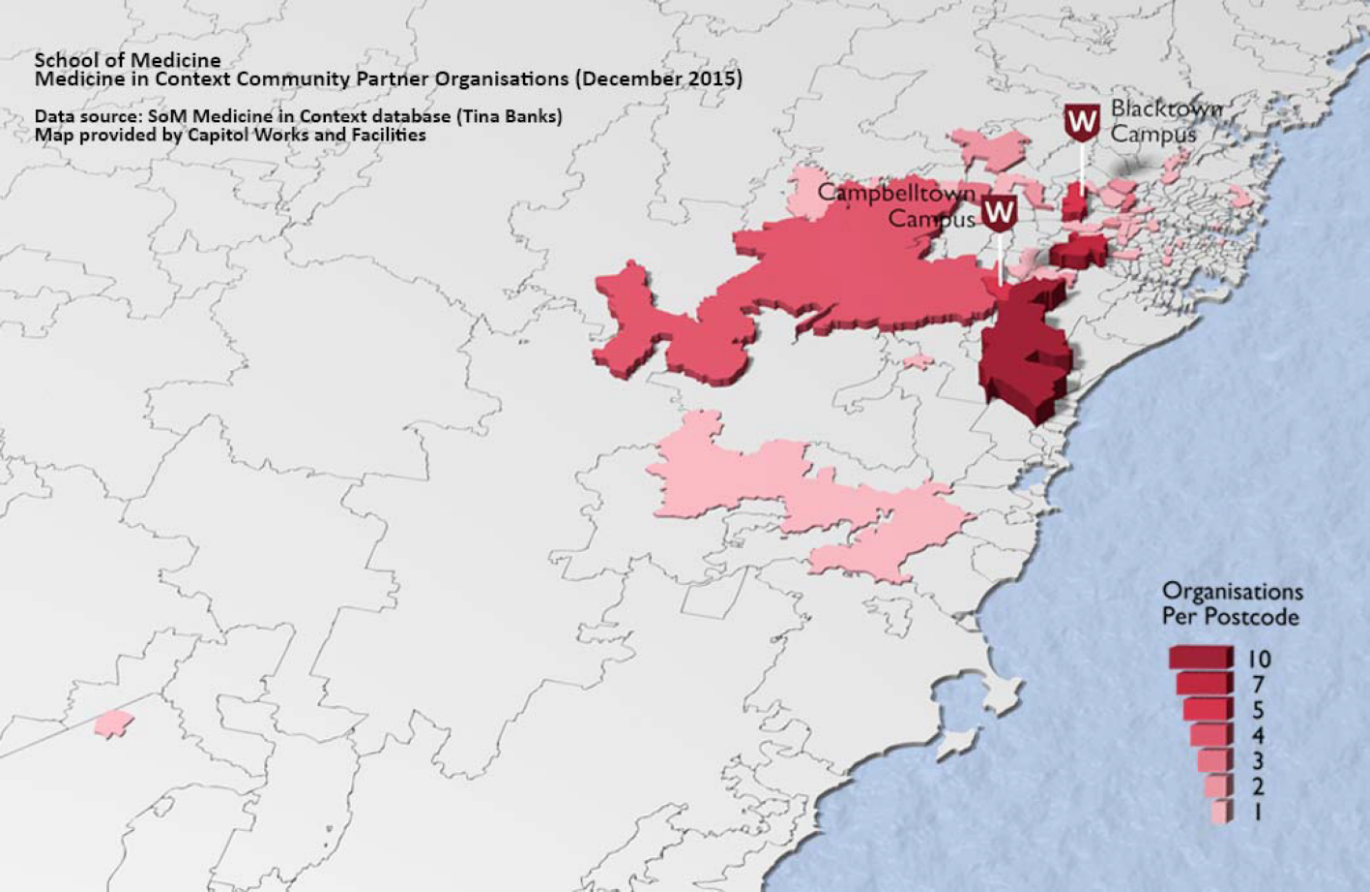
Geographical distribution of the WSUSoM Medicine in Context community partner organisations in 2015.

To date, the WSUSoM has partnered with over 100 community organisations as MiC placement hosts at community-based services in: Aboriginal and Torres Strait Islander health, aged care, children and young people, community care, disability, drugs and alcohol, health promotion, men’s health, mental health, refugee and migrant health, sexual health, and women’s health. Students are exposed to diversity through immersion with service providers and their clientele. Most community partners serve the GWS region but some are located in other parts of Sydney and outside Sydney such as in the Australian Capital Territory (see figure 2 (Fig. 2)). This geographic spread enriches the variety of community settings and demographics where students learn. 

MiC community partners introduce students to a broad range of health services. For example, students may attend a home visit for a mother with postnatal depression, visit a post-stroke client who requires home modifications, or participate in a multicultural day health check for seniors. Students are encouraged to learn about the services and observe service providers’ communication styles when engaging with different cultures, diverse health literacy levels, and challenging clients. Community placement supervisors regularly debrief students to address any concern and discuss how they may incorporate new insights into clinical practice. Students are guided to view patients holistically through discussions about social determinants of health and their influence on patients’ health outcomes.

##### 2.2.2. GP placements

**Figure 3:**
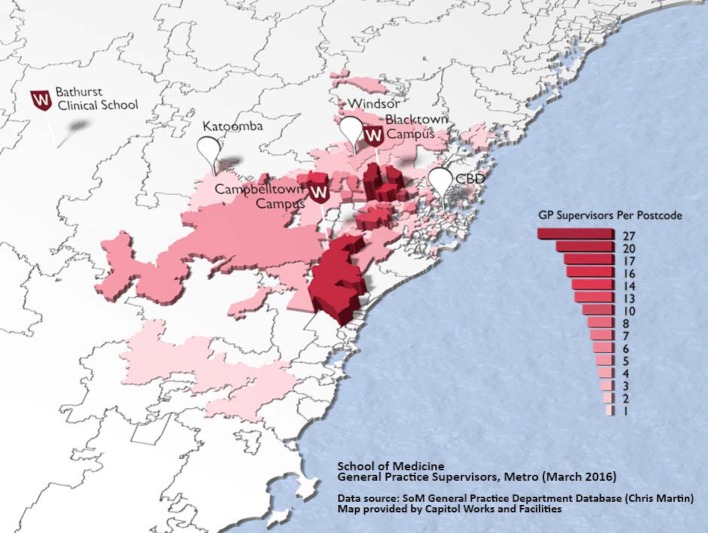
Geographical distribution of the WSUSoM Medicine in Context GP partners in 2016.

MiC provides students with their first exposure to GP clinics, which forms a foundation for further GP learning in the final year in a vertical curriculum integration. MiC GP placements mirror the community placements’ geographical spread and diverse clientele (see figure 3 (Fig. 3)). Students are directly exposed to the delivery of primary care to people from different cultures, local epidemiology, and levels of socio-economy, health literacy and English proficiency. Students must demonstrate their learning across each of the Five Domains of General Practice as determined by The Royal Australian College of General Practitioners (RACGP), the national accrediting body [17]. Students are assessed by their GP supervisors on their understanding and performance in all of the five domains. Diversity education in MiC particularly supports two of the five RACGP domains i.e. “Population health and the context of general practice” (which covers epidemiology, public health, prevention, family influence on health, resources) and ‘Communication skills and the patient-doctor relationship’ (which covers communication skills, patient-centredness, health promotion, whole person care). 

##### 2.2.3. Tutorials

Students share and synthesise their learning every week in tutorials. MiC tutors come from diverse professional backgrounds (sociology, psychology, chiropractic, medicine, education, nursing, chaplaincy) and life experiences (age, ethnicity, migration, religion/spirituality, disability, gender and sexuality). With students coming from a multitude of backgrounds and being placed in various community and GP settings, MiC tutorials provide an opportunity to dissect MiC learning experiences from various angles which enriches students’ learning. For example, a tutorial discussion on trends in mental health saw students sharing their culture’s acceptance or rejection of the concept of mental health, thus exposing the fundamental challenges in designing preventative strategies aimed at the general community. 

##### 2.2.4. Workshops

Community supervisors share their insights of diversity in providing their services in a series of MiC workshops on campus. These workshops raise discussions around managing diversity such as: social class, family structure (including same-sex parents), ethnicity, language barriers, cultural identities, racism, health literacy, gender and sexuality, domestic violence, health-seeking behaviours, and disability. All workshops are co-facilitated by MiC academics and community service providers and the case studies are taken from placement organisations’ real clients. Community partners also contribute their resources such as video clips made by non-verbal clients with disability using communication aids. Clinical aspects are integrated in all cases to train students in tailoring routine clinical tasks to the specific contexts of patients from diverse backgrounds, such as in breaking bad news, identifying domestic violence, discussing complementary medicine use, managing augmentative and alternative communications, contact tracing of sexually transmissible infections, and managing medications and referral pathways. The last workshop in the series discusses three patients with complex cases: drug addiction with domestic violence; autism spectrum disorder with XXYY chromosome; and paranoid schizophrenia with a court restraining order. This final workshop brings together the students’ learning and highlights the intersections of multiple diversities in clinical settings.

#### 2.3. Clinical integration

Supervisors, tutors and workshop facilitators make a concerted effort to link students’ MiC diversity education to their clinical learning. All discussions and sharing of insights are eventually brought to a question about how students can use their community-based diversity learning in clinical settings. Clinical integration of MiC diversity learning is also supported by assessments. Students are required to write an academic essay on a social determinant of health of their choice in light of all of their MiC experiences. The MiC team including community partners contribute to the end-of-year written exams. Since 2015 MiC has been regularly incorporating diversity aspects such as disability, social isolation, culture, and health literacy into the WSUSoM standardised clinical examination system (Objective Structured Clinical Examinations). 

Below are some examples of integration between diversity and clinical learning in MiC.

##### 2.3.1. Disability service placements 

Disability service placements help students learn about physical and cognitive disabilities in social and healthcare environments with experienced allied health professionals. Students learn about provision of support in complex human service environments, disability factors which may obscure clinical diagnoses [18], and self-determination, community participation and inclusion within the health context and wider community. Students are facilitated to transition from a medical model where individuals are defined by their deficits to a social model where disability is recognised as an intersection between the individual’s impairments and the barriers created by society [19]. Students also discuss strategies to advocate for more inclusive medical and social environments for people with disabilities.

##### 2.3.2. Community health centre placements 

Community health centre placements expose students to services that meet the specific needs of individuals, families and community groups including vulnerable population groups. The diversity of services offered include medical and nursing, allied health, counselling, health promotion, aged care, drugs and alcohol, maternal and child health, mental health, and disability. Students are asked to reflect on how they see their future role as a doctor in relation to these services. Some students have critically questioned the vulnerable communities' ability to navigate the increasingly complex health services and digital/online health information and communication.

##### 2.3.3. Aged care placements 

Aged care placements enable students to experience diversity within the 60+ age group. Communicating with and observing people affected by social isolation and dementia has resulted in reflective conversations on how the experience will affect students’ future medical careers. The realisation that social contact can increase individuals’ health and wellbeing is a powerful learning experience for students. Clinically, aged care placements also introduce students to the various clinical presentations of dementia. Several students were surprised to experience the difficulty in recognising dementia in some patients with whom they had daily contact, which gave them first-hand insight into the challenge for clinicians in identifying dementia during brief clinical encounters. 

#### 2.4. Evaluation

All MiC components are evaluated at the end of each 5-week block. Students complete evaluation questionnaires about their community and GP placements, tutorials, and workshops. Community and GP supervisors assess their students and provide evaluation in placement assessment forms, where anecdotes such as reflective discussions were reported. Students’ essays and their performance in MiC-related exam questions are also monitored by the MiC Team, and the findings are qualitatively incorporated to the overall MiC program evaluation. All evaluation data are analysed by the MiC Convenor and aggregated reports are distributed annually to placement supervisors, the WSUSoM committees and student representatives. Any action taken based on evaluation data is reported in MiC curriculum documents in the following year. 

Students' evaluation scores and comments, performance in assessments, and alumni feedback on the value of MiC have indicated a strong ongoing value in medical application from this program. From time to time there has been resistance from some students particularly when they could not see the clinical relevance of their community-engaged diversity education. The MiC Team engages student representatives including those who expressed dissatisfaction in designing improvements in the program, which has resulted in a diminishing number of complaints. Another regular challenge has been the need to balance between standardising students’ learning and maintaining the unique experiences offered by each placement. The MiC learning outcomes and their associated assessment criteria form the parameters to which each placement learning activity need to adhere; and there is ample room within those parameters to accommodate site-specific placement learning activities. These learning activities and their alignment to the MiC learning outcomes and assessment criteria are monitored through MiC Placement Learning Plan documents which are discussed between students and their supervisors.

#### 2.5. Transition 

Since 2016, steps have been taken to remodel the MiC program in line with the WSUSoM transition from MBBS to MD in 2019-2023. Extensive consultation involving individual and small group meetings with community partners and student representatives were conducted in 2017-2018, and continues as the new curriculum is being rolled out. The MD MiC program will be better integrated vertically across the five years, and horizontally by creating new intersections with population health, problem-based learning, general practice and hospital-based rotations (see table 1 (Tab. 1)). Students’ exposure to diversity in GWS and beyond will be expanded through 2-year MD Scholarly Projects which include community-based research and service learning options. 

## 3. Results and discussion

Multifaceted diversity education is at the forefront in MiC pedagogy and curriculum contents. First, MiC content was developed based on the health priorities which are grounded on community voices and contemporary challenges in the highly diverse GWS population. The structure and delivery of the MiC program enables students to understand the health system and the interplay between general practice and referral networks that connect individuals to community services that support their needs. Community immersion hones the importance of multidisciplinary care, and how community organisations can support general practitioners and specialists. Diversity education in MiC thus fosters a broader perspective in medical students that is beneficial in delivering effective patient management. The teaching also provides students with the tools and knowledge to manage a patient’s background appropriately and effectively. Through interactions with people from different backgrounds, students learn about effective communication, patients’ journey through the health system, and the importance of gaining optimal clinical outcomes that respect the patients’ health needs, wants and influencers.

Second, the MiC program acknowledges the diversity among students, and is designed to allow each student to craft their unique understanding of how social determinants of health impact themselves and their communities. Case studies involve patients/clients from diverse ethnic, gender and socioeconomic backgrounds which support the students who may identify with some of these markers. A multitude of teaching methods (games, group works, role plays) enable students to collectively participate thus increasing their engagement. By working in small groups and cultivating an atmosphere of respect, MiC tutorials provide a safe space for students to reflect upon their backgrounds and values and to celebrate and discuss their individual diversity [1]. MiC also uses several assessment modalities which enable students with different learning strengths to demonstrate their achievements. Reflection on teaching methods with an ongoing commitment to evaluating student learning experiences has resulted in the use of contemporary approaches to meet student learning needs, such as replacing didactic lectures with flipped classrooms [15]. 

Third, from the clinical perspective, the MiC program increases students' awareness of the impacts of social determinants of health on patient health outcomes [20]. Student learning about diversity is integral to clinical learning given the diversity of patients in age, ethnicity, socio-economic status and health issues encountered on a daily basis, particularly in regions such as GWS. The exposure to diversity helps students place their clinical learning within the broader patient context. The MiC program, undertaken within and in partnership with the community during the initial clinical year, is uniquely placed to challenge future doctors to look beyond stereotypes and adopt a sense of social accountability [21]. 

Fourth, the MiC diversity education improves students' literacy about community-based services. Given that patients encountered in clinical settings, particularly in GP, may need to access community-based services, it is imperative that students gain insight into how these services support the patients, and the inter-professional dynamics between health and non-health professionals. The widespread MiC community and GP placements expose students to a range of patients’ lived realities, which demonstrates to students that causes for poverty and inequities in health and access to healthcare are as numerous as the patients that they see [20].

Fifth, the MiC team was deliberately constructed to include diverse professionals from within and outside the WSUSoM (as reflected in the authorship of this paper) to expand students’ perspectives during their learning and to model the collaboration students will need to establish in their medical careers. The team share not only their professional insights but also personal insights when relevant, such as first-hand accounts about the health and social challenges in caring for family members with disabilities, being a migrant, or being same-sex attracted. The MiC team also offers support for students who are facing challenges related to their identification with certain aspects of diversity such as disability or non-white ethnicity. 

The MiC team has encountered resistance from some students that hampered their engagement with the MiC learning process. Such resistance is commonly found in diversity education [22] and has a distinct impact on patient engagement and community help-seeking behaviour [23]. Since diversity education is integral to improving health outcomes and health system work cultures [23] as desired by the community, the MiC team work with student resistance by highlighting the relevance of diversity education to clinical learning. The MiC team members are careful to address any discomfort students may have in addressing diversity [24], and discourage students from being ‘blind’ to differences [25]. Exposure to and engagement with diversity in MiC is important for students to become comfortable with being uncomfortable, break down resistance and effectively work through challenges to learning about diversity [1]. A formal evaluation research on MiC is underway, and ongoing qualitative and quantitative reviews as part of MiC quality assurance have identified improvements in students' quality of learning and satisfaction with the program particularly over the past four years [14], [15]. Reflective post-placement conversations between students and supervisors identified that students had grown personally and professionally by addressing challenges identified at the beginning of their placements, with an increased understanding of the diverse community in which they will practice.

## 4. Conclusion

The the WSUSoM MiC program – developed for over ten years in collaboration with community and GP partners – exposes medical students to diversity. The program has successfully implemented community-engaged learning and partnership pedagogy to prepare medical doctors with insights into their patients’ complex world outside the hospital. In increasingly diverse societies like Australia, such learning is key to developing competency in patient-centred care.

## Competing interests

The authors declare that they have no competing interests. 

## Figures and Tables

**Table 1:**
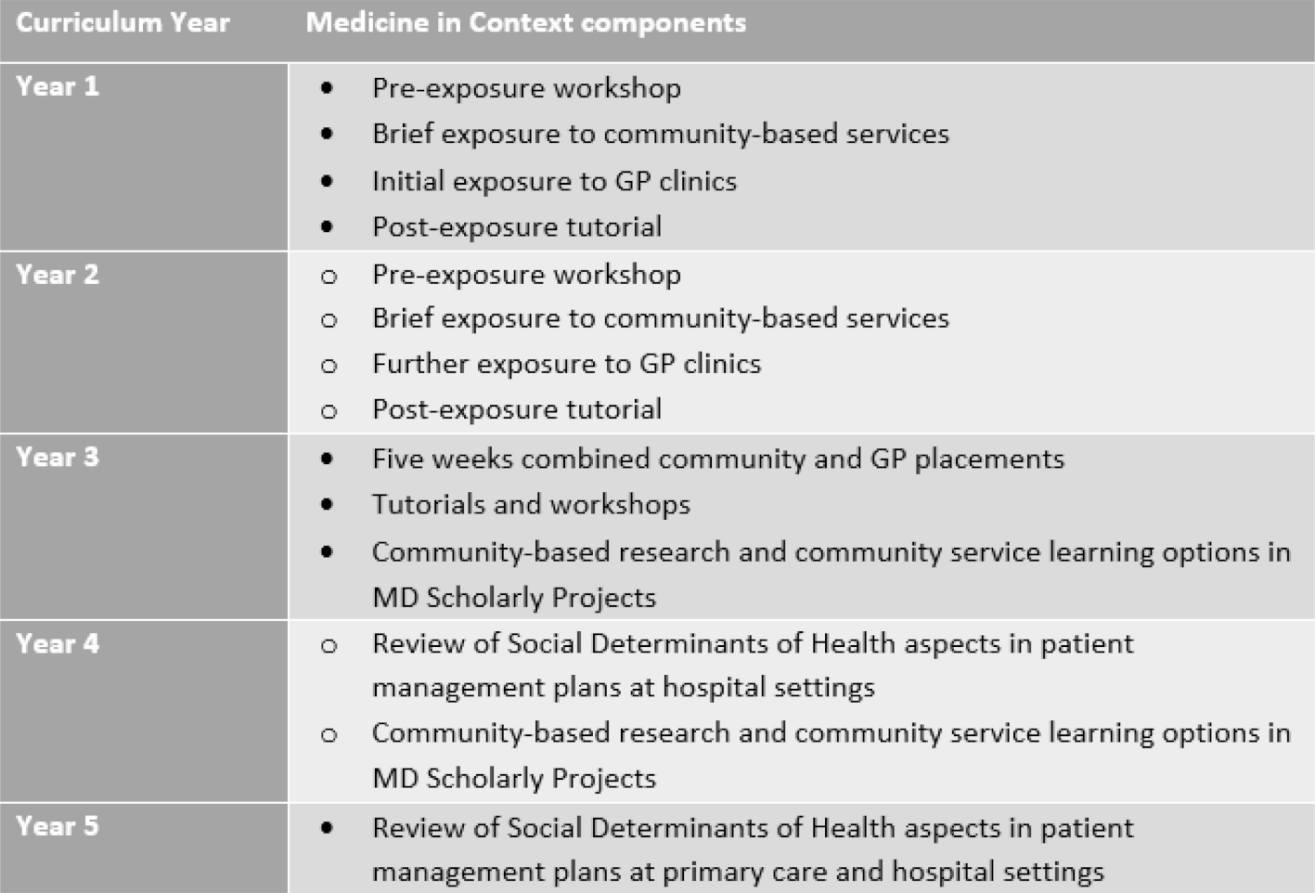
Medicine in Context program location and structure at the Western Sydney University School of Medicine MD curriculum.
